# Editorial: Nature and the Environment: The Psychology of Its Benefits and Its Protection

**DOI:** 10.3389/fpsyg.2015.01804

**Published:** 2015-11-25

**Authors:** Daniel J. Hayes, Marc G. Berman

**Affiliations:** Department of Psychology, The University of ChicagoChicago, IL, USA

**Keywords:** nature, climate change, benefits of natural environments, urban design, attention restoration theory

The goal of this Research Topic was to bring together research and scholarship from two seemingly disparate fields: (1) the psychological and health benefits attained by interacting with natural environments and (2) the variables that facilitate people's recognition of environmental issues that would foster a more positive attitude toward the protection of nature.

The first portion of this e-book concerns the positive benefits (health, psychological, affective, etc…) that can be gained from interacting with more natural environments. There is now a considerable amount of evidence in support of the beneficial effects that nature can have on an individual's cognitive functioning and health. Kuo (2015) provides an excellent review toward understanding he interaction of nature and health. Kuo hypothesizes a central pathway for how nature improves health through enhanced immune functioning. Furthermore, Kuo suggests public policy to implement green spaces with plants, soil, and moving water in areas where health risks are high as an inexpensive public health intervention.

Szolosi et al. (2014) examined if nature's perceived mysteriousness had an effect on direct cognitive benefits. They showed that with enough exposure to an image, more mysterious nature images achieved greater improvements in recognition performance than nature images with low perceived mystery (Szolosi et al., 2014). Capaldi et al. (2014) performed a meta-analysis regarding the potential beneficial relationship between nature and health. Reported vitality, positive affect, and life satisfaction all had a strong relationship with nature connectedness. This analysis further concluded strong associations between happiness and inclusion of nature in the self, nature relatedness, and connectedness to nature (Capaldi et al., 2014). Interestingly, participants do not necessarily have to be consciously aware of the restorative environmental stimuli to obtain the benefits. According to the results of Lin et al. (2014), participants received an effect of attention restoration with minimal or absent awareness to the restorative stimuli using digital images. We must also be cautious when it comes to some of this environmental research. Pearson and Craig (2014) performed a review of the existing literature and call for future research to focus on substantiating the rather simplistic dichotomy of “nature” vs. “built” environments (Pearson and Craig, 2014). The review by Pearson and Craig also brought up the topic of immersive of the nature intervention. Many studies have focused primarily on studying human interactions with only images of natural and urban environments. The authors suggest that future studies should explore different modes of immersion (e.g., virtual realities, enhanced means of exposure) when studying environmental impacts on psychological and physical health.

In addition to this expanding upon this simplistic dichotomy, it may also be important to examine differences in culture and urbanization (level of immersion in urban environments). Linnell et al. (2014), compared concentration abilities in two different cultures that reside in environments that have vastly different urbanization levels (Linnell et al., 2014). They found that urbanization can lead to a reduced ability to concentrate, as measured with a line bisection task, compared to individuals who reside in a less urbanized society.

In addition to documenting the differential benefits of nature, it is also important to try to understand what is it about nature itself that leads to these benefits. As previously stated, researchers have found beneficial effects simply from viewing pictures of nature. This suggests that there may be low-level visual features within natural environments that may lead to psychological benefits. Kardan et al. (2015) found that color-related and edge-related characteristics of nature interactively contribute to preferring one scene to another (Kardan et al., 2015). Spatial structure and color properties were quantified; then, nature and man-made environments were decomposed after being rated on subjective preference and naturalness. Multiple regression analysis showed that some of these features could significantly predict preference of images: straight-edge density, lower hue level, and higher diversity in saturation. A separate study, integrates design aesthetics, mathematics, and recent psychology theories (Hunter and Askarinejad, 2015). From this integration of methodologies, the authors developed a list of physical attributes that explained image features within the environment (e.g., horizon line, building distribution, natural phenomena). These attributes provide even more insight into objective environmental features that may explain why cognitive restoration occurs in natural environments. Furthermore, this research may aid in the design of urban areas to optimize the restorativeness of the built environment.

The second portion of this e-book centers on cutting-edge research for how to educate the general public on environmental issues, as well as public policy implementation. Jacquet et al. (2014) investigated motivational bases of environmental attitudes and behaviors by focusing on specific ideological factors, in addition to general psychological principles. The authors devise a dichotomy of mutually reinforcing influences: top-down (e.g., corporate strategy, mass media, and political discourse) and bottom-up (e.g., ego, group, and system justification). These influences converge at an ideological divide over climate change. The authors conclude that regardless of the influence, future messages must allocate more attention to how they are framed and delivered to the public. Another dichotomy exists between two moral attitudes, both pertaining to concerns for the environment: anthropocentric and biocentric (Rottman, 2014). Anthropocentric, a word which its prefix means “human” refers to the concerns aimed at preserving the welfare solely for humans. It is within the moral attitudes of biocentrism that a crucial environmental distinction should be made between harm and purity, particularly because it extends mental states and rights to non-human entities unlike anthropocentric attitudes. The study of these distinctions could provide moral clarity to issues such as greenhouse gas emissions and an end to deforestation. There is an even more pressing need to make individuals more proactive in protecting and conserving the environment. De Young (2014) posits that biophysical restraints, such as dwindling natural resources are not problems that can be solved; rather, they are complex predicaments that must be endured. The author praises the small experiment framework for its pragmatic exploration into useful environmental-person interactions, and calls for a development school of biophysical psychology to investigate ways to make individuals more accepting of policy measures to mitigate climate change. A concurrent review by Page and Page (2014) used a novel framework to create alternative perspectives on variables impacting pro-environmental activity and behavioral change. These variables can be later compared with other psychological variables derived from alternative theories of behavior. This research topic recognizes the eclectic contributions from mathematics, psychology, and public policy to encourage: proactive behaviors to preserve the environment; and research to continue investigating how and why interacting with natural environments are beneficial to both physical and psychological health.

## Author contributions

DH and MB wrote the paper.

### Conflict of interest statement

The authors declare that the research was conducted in the absence of any commercial or financial relationships that could be construed as a potential conflict of interest.
